# Cross-validation of analytical methods for citrate ligand quantification on upconversion and iron oxide nanoparticles

**DOI:** 10.1007/s00216-026-06402-8

**Published:** 2026-03-04

**Authors:** Anna Matiushkina, Sven Brehme, Isabella Tavernaro, Elina Andresen, Sarah-Luise Abram, Matthias Koch, Ute Resch-Genger

**Affiliations:** 1 Division Biophotonics, Bundesanstalt für Materialforschung und -prüfung (BAM), Richard-Willstaetter-Straße 11, 12489 Berlin, Germany; 2 Department of Biology, Chemistry, and Pharmacy, Free University Berlin, Arnimallee 22, 14195 Berlin, Germany; 3 Division Technical Properties of Polymeric Materials, Bundesanstalt für Materialforschung und -prüfung (BAM), Unter den Eichen 87, 12205 Berlin, Germany; 4 Division Organic Trace and Food Analysis, Bundesanstalt für Materialforschung und -prüfung (BAM), Richard-Willstaetter-Straße 11, 12489 Berlin, Germany

**Keywords:** Upconversion nanoparticles, Iron oxide nanoparticles, Citrate ligand quantification, High-performance liquid chromatography, Thermogravimetric analysis, Pyrolysis-gas chromatography-mass spectrometry

## Abstract

Analysis of nanomaterial (NM) surface chemistry is essential for understanding and controlling NM properties relevant for NM functionality, stability, and safety. In this work, we present a multimethod study of the quantification of the frequently used, biocompatible surface ligand citrate for lanthanide-based upconversion nanoparticles (UCNPs) and iron oxide nanoparticles (IONPs). Methods applied that address different yet correlated measurands with varying chemo-selectivity and rely on different sample preparation workflows include chemo-selective reversed-phase high-performance liquid chromatography (RP-HPLC) with photometric detection measuring liquid samples and dissolved NMs as well as non-selective thermogravimetric analysis (TGA) and chemo-selective pyrolysis-gas chromatography-mass spectrometry (PyGC-MS) analyzing dried NMs. Thereby, we demonstrate the applicability of the RP-HPLC method, recently developed for citrate quantification on IONPs, for UCNPs as another application-relevant NM class. In addition, the feasibility of PyGC-MS, emerging for material characterization at the trace level due to the consumption of very small sample amounts and minimum sample preparation, for NM ligand quantification is assessed. This method, which has not been applied before for NM surface analysis, indirectly quantifies citrate via the amount of acetone formed during citrate decomposition. For UCNPs, PyGC-MS measurements led to a good agreement with the TGA and RP-HPLC results within the respective measurement uncertainties. However, quantifying citrate on IONPs with PyGC-MS resulted in an overestimation of citrate, which is tentatively ascribed to IONP catalytic activity. Overall, our method cross-comparison underlines the importance of multimethod characterization schemes to assure a reliable and precise ligand quantification and to identify method- and NM-specific effects.

**Graphical abstract:**

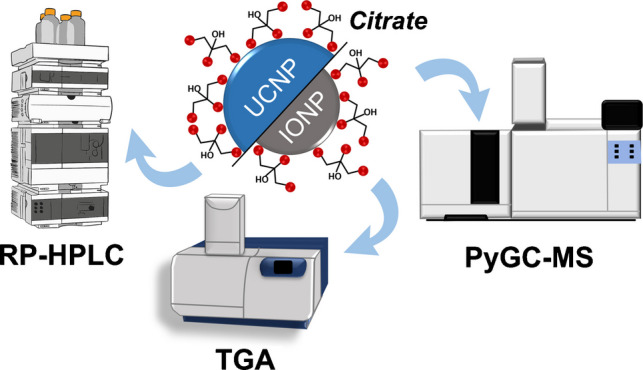

**Supplementary Information:**

The online version contains supplementary material available at 10.1007/s00216-026-06402-8.

## Introduction

In the last years, inorganic and organic nanomaterials (NMs) have been increasingly exploited in the material and life sciences, with applications, e.g., in medicine [1–4], energy technologies [5, 6], optoelectronics [7], and catalysis [8]. These NMs typically present core or core/shell nanostructures, which are electrostatically and/or sterically stabilized with covalently or coordinatively bound surface ligands. Their functionality and hence their suitability for specific applications are largely determined by size, morphology, chemical composition, and crystal phase, while their colloidal stability, dispersibility, processability, and interaction with the surroundings and biological species are mainly controlled by their surface chemistry [9, 10]. This triggered the development of methods for controlled NM surface functionalization and modification [11–14] and the determination and quantification of surface ligands and functional groups (FGs) [9, 15, 16] [17, 18]. The recognition and increasing awareness of the need to accurately characterize NMs during production and for risk and toxicity studies require not only the measurement of structure-analytical physicochemical NM properties, but also call for reliable and validated tools for surface analysis [16, 19]. This fuelled current activities on standardizing methods for NM surface analysis and the development of test and reference materials for method validation and instrument performance control [20]. Information on surface chemistry is also important to derive property-application and property-safety relationships for NM grouping, and safe and sustainable by design (SSbD) NM concepts [10].

Meanwhile, many analytical methods have been developed to determine and quantify NM surface FGs and ligands that differ in measurand, signal generation principle, need for a signal-generating reporter, which can vary in size, spatial requirement, and charge, as well as chemo-selectivity, quantification potential, and sensitivity or limit of detection (LOD) [11, 15, 16, 19]. Also, sample preparation workflows, instrument complexity and costs, time consumption, and ease of automation can substantially vary [11, 15, 18, 21]. Methods explored include quantitative nuclear magnetic resonance (qNMR) techniques [22–25], X-ray photoelectron spectroscopy (XPS) [26], vibrational spectroscopy [27], thermogravimetric analysis (TGA) [28], and electrochemical titration techniques [9, 18, 29]. Also, simple optical assays are often used, particularly by NM manufacturers for process and quality control, which require a chemical reaction or an electrostatic interaction of the FGs and the signal-generating reporter [29, 30].

Our interest in developing methods for NM surface analysis varying in measurand, detection principle, chemo-selectivity, sample preparation workflows, and throughput as well as automation potential encouraged us to explore the applicability of established analytical techniques from organic and inorganic chemistry for NM ligand analysis. In this respect, in addition to methods such as optical assays, electrochemical titration methods, qNMR, and XPS [17, 18, 21, 23], we recently explored the suitability of high resolution-continuum source-graphite furnace molecular absorption spectrometry (HR-CS-GFMAS) for the surface analysis of aminated silica nanoparticles and microparticles labelled with optically detectable fluorine containing reporters [31]. A particularly interesting ligand presents citrate, which is frequently employed for stabilizing metal, metal oxide, and lanthanide nanoparticles (NPs) in hydrophilic environments [32, 33]. Advantages of citrate are its biocompatibility and simple replacement by other more strongly binding ligands in post-synthetic surface modification reactions [13, 34]. Although citrate is frequently measured in medical and food analysis [35, 36], its quantification on NMs has been rarely explored. Examples include the determination of citrate on metal and metal oxide NPs using TGA, elemental (CHN) analysis, and Fourier transform infrared spectroscopy (FTIR) [37–40]. We recently examined and demonstrated the citrate quantification potential of reversed-phase high-performance liquid chromatography (RP-HPLC) with photometric detection for representatively chosen iron oxide nanoparticles (IONPs) as well as solution qNMR [41]. The latter required complete removal of (para)magnetic iron species.

**Fig. 1:**
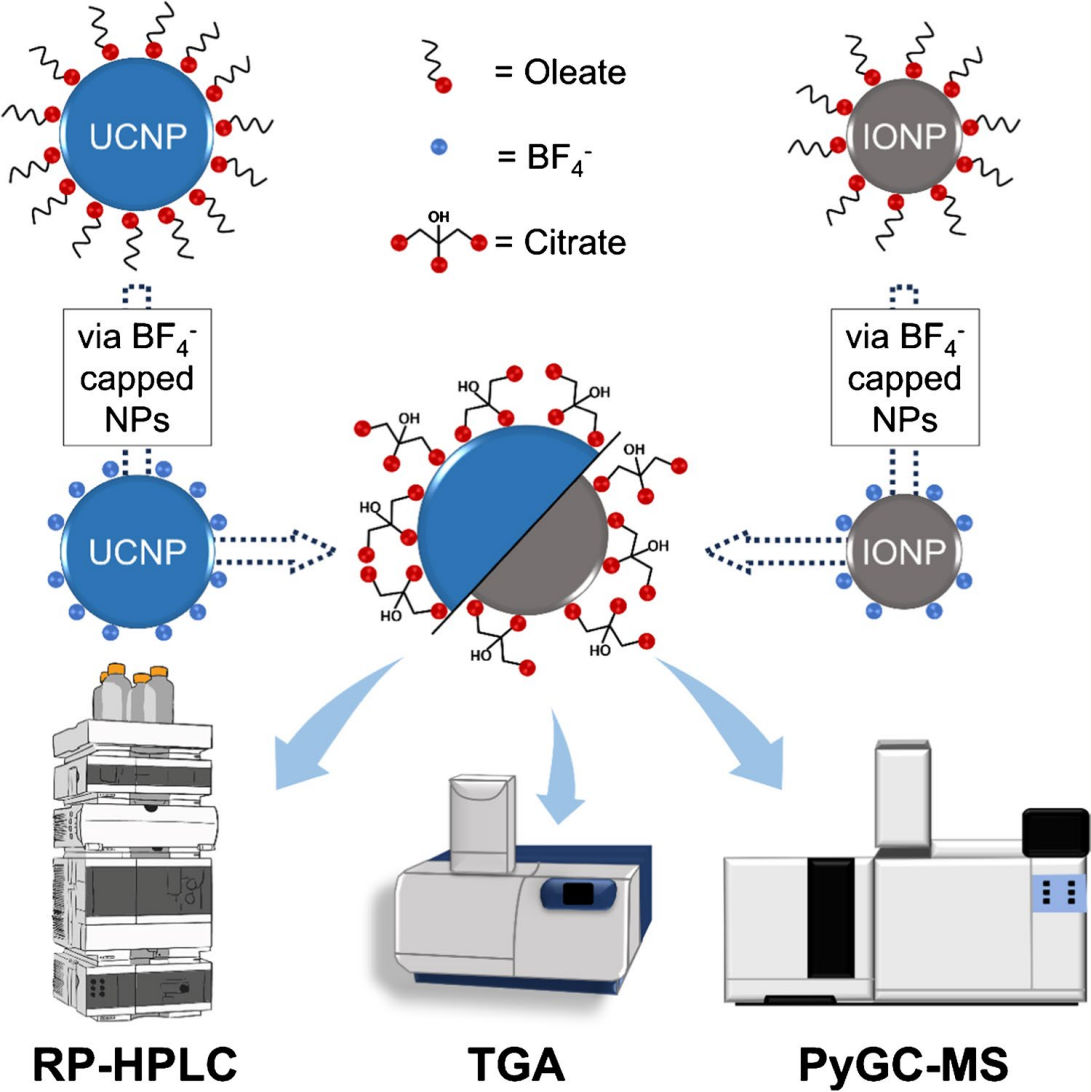
Schematic overview of the multimethod approach to quantify citrate on the surface of representatively chosen UCNPs employing previously validated chemo-selective RP-HPLC with photometric detection for dissolved NM samples as well as non-specific TGA and chemo-selective PyGC-MS for dried NM samples. To assess and identify possible NM-specific effects on PyGC-MS measurements in this first proof-of-concept study, where citrate is indirectly quantified via its decomposition product acetone, we subsequently included IONPs in the study, using a similar surface modification workflow as employed for the preparation of citrate-capped UCNPs

In the present study, we demonstrate the versatility of our novel RP-HPLC method with photometric detection for quantifying citrate on surface-functionalized NMs, here for luminescent lanthanide-based upconversion NPs (UCNPs; NaYF_4_:Yb^3+^,Er^3+^), selected for their increasing usage in bioimaging, medical diagnostics, and material sciences [42]. Citrate surface modification of the initially hydrophobic UCNPs, prepared by the established thermal decomposition method in the presence of oleic acid [43], involved a two-step procedure, i.e., the removal of oleate ligands by NOBF_4_ followed by introducing citrate [43, 44]. This ligand exchange workflow differs from the surface modification procedure used for our multimethod study of citrate-stabilized IONPs with RP-HPLC and qNMR spectroscopy, employing method cross-comparison for method validation [41]. However, the surface chemistry of the as-prepared UCNPs and IONPs is similar with oleate capping ligands that may not be fully replaced by citrate. In addition to our citrate-selective RP-HPLC method, we utilize non-specific TGA, measuring the mass loss during successive heating, and assess the quantification potential of another chromatographic method, pyrolysis-gas chromatography-mass spectrometry (PyGC-MS). PyGC-MS enables the analysis of very small amounts of solid or liquid samples without sophisticated sample preparation steps, making it particularly attractive, e.g., for polymer identification and quantification [45, 46], including targeting plastic pollution and microplastics in environmental matrices [47–49]. However, this method has not been employed before for the analysis and quantification of surface ligands on NMs. This encouraged us to explore the applicability of PyGC-MS for this increasingly important area of application in a proof-of-concept study. An overview of the study including NMs and methods involved is given in Fig. 1.

## Experimental

### Materials

Sodium hydroxide (NaOH, ≥ 99.5%), iron(III) chloride hexahydrate (≥ 99%), ethanol absolute (≥ 99.9%), *N,N*-dimethylformamide (DMF, ≥ 99.9%), glacial acetic acid (≥ 99.5%), hydrochloric acid (HCl, 37%), methanol (gradient grade for HPLC, ≥ 99.85%), chloroform (≥ 99.5%), absolute ethanol (EtOH, ≥ 99.8%), cyclohexane (≥ 99.5%), 1,2-dichlorobenzene (≥ 98%), diethyl ether (≥ 99.5%), and acetone (≥ 99%) were purchased from Chemsolute (Th Geyer, Germany). Yttrium(III) chloride hexahydrate (99.9%), ytterbium(III) chloride hexahydrate (99.9%), erbium(III) chloride hexahydrate (99.9%), sodium oleate (≥ 82%), oleic acid (90%), phosphoric acid (85%), and sodium hydroxide solution (NaOH, 1 N) were purchased from Sigma-Aldrich (Merck, Germany). n-hexane (≥ 99%) was obtained from AppliChem GmbH (Germany), 1-octadecene (ODE, 90%) from ThermoFisher Scientific, ammonium fluoride (NH_4_F, ≥ 98%) from Honeywell, nitrosyl tetrafluoroborate (NOBF_4_, 98%) from abcr GmbH (Germany), trisodium citrate dihydrate (99%) from Alfa Aesar, sodium citrate tribasic dihydrate (≥ 99.5%) from Fluka, sodium dihydrogen phosphate dihydrate (≥ 99%) from Carl Roth GmbH (Germany), and citric acid (99%) from Glentham Life Sciences Ltd. (United Kingdom). All chemicals were used without further purification. All aqueous solutions and buffers were prepared with ultrapure water (0.055 μS·m^−1^; MilliQ water, Merck Milli-Q® IQ 700).

### Synthesis of UCNPs and IONPs and ligand exchange procedures

Co-doped oleate-capped NaYF_4_:Yb^3+^,Er^3+^ UCNPs were synthesized following a procedure from Wilhelm et al. reported for the large-scale synthesis of hexagonal-phase UCNPs [43]. To prepare hydrophilic citrate-capped UCNPs from the initially oleate-stabilized hydrophobic UCNPs, a two-step ligand exchange process was used [43]. The first step involved the removal of the hydrophobic oleate ligands in a biphasic system of cyclohexane and DMF under stirring at room temperature (rt, T = 23 °C) in the presence of NOBF_4_, which generates a mildly acidic environment to promote ligand detachment, thereby transferring the UCNPs from the apolar cyclohexane into the polar DMF phase. As the second step of the ligand exchange procedure, the UCNPs redispersed in DMF were mixed with an aqueous solution of trisodium citrate dihydrate under stirring at rt to introduce citrate as a hydrophilic capping agent stabilizing the UCNPs in aqueous solution. The resulting citrate-capped UCNPs were dispersed in MilliQ water and stored at 4 °C.

Oleate-capped IONPs were synthesized following the procedure from Park et al. [50], exploiting the thermal decomposition of iron oleate in ODE with oleic acid at 320 °C under an argon flow. To exchange oleate for citrate ligands, for the IONPs, a procedure similar to the ligand exchange employed for the UCNPs was used with minor changes. The resulting citrate-capped IONPs were dispersed in MilliQ water and stored at rt.

For a direct ligand exchange procedure [34], which was also previously used by us [41], the oleate-capped IONPs were redispersed in 1,2-dichlorobenzene, mixed with DMF containing citric acid, and stirred at 100 °C for 24 h. The resulting citrate-capped IONPs (IONPs-2) were dispersed in MilliQ water with the addition of NaOH solution and stored at a pH of 7.9 at rt.

The detailed ligand exchange procedures are given in the Supporting Information (SI).

### Nanoparticle characterization

NPs were characterized by dynamic light scattering (DLS) and zeta potential measurements at neutral pH using a Zetasizer Nano ZS (Malvern Panalytical Ltd.), equipped with a 633 nm laser. DLS measurements were performed in duplicates for NP dispersions with concentrations of about 1 mg/mL at 25 °C. For DLS measurements, a back scattering detection angle of 173° was used. The hydrodynamic diameter was derived with the cumulant method and calculated for a number-based distribution (d_h,0_). The zeta potential was determined based on the electrophoretic mobility of NPs using the Smoluchowski model. DLS and zeta potential measurements were analysed using refractive indexes of 1.475, 2.800, and 1.330 for UCNPs, iron oxide, and water, respectively, as well as a viscosity of 0.8872 cP for water.

Transmission electron microscopy (TEM) measurements were carried out using a Talos F200S microscope (Thermo Fisher Scientific) with an accelerating voltage of the electron beam of 200 kV. The samples were prepared on carbon-coated copper grids (Plano GmbH) by drop casting and drying at rt. The obtained TEM micrographs were analysed using the software ImageJ (Version 1.54 g), evaluating around 100 and 30 particles for citrate-capped UCNPs and IONPs, respectively.

### Citrate quantification

TGA measurements of previously dried NM samples were performed with a Hitachi STA 7200 setup with an AS3 Sample Charger. Thermogravimetric (TG) curves and the corresponding derivative thermogravimetric (DTG) curves for citrate-capped NPs were recorded in duplicates under argon atmosphere at a heating rate of 10 °C/min.

RP-HPLC experiments were performed in triplicate using a 1260 Infinity system from Agilent Technologies equipped with a diode array detector (DAD). An Eurosphere II 100-5 C18 (250 × 4.6 mm) column was used, the injection volume was 20 µL, the flow rate 1 mL/min, and the column temperature 30 °C. The mobile phase consisted of A: a phosphate buffer (pH 2.9 ± 0.1; 0.5% sodium dihydrogen phosphate solution, pH adjusted with 85% phosphoric acid) and B: methanol, utilized in an isocratic ratio of A:B = 97.5:2.5. The signal was recorded at 210 nm at the absorption maximum of citric acid. Samples were prepared by dissolving 90 µL of a NM stock dispersion with 200 µL of HCl (37%) followed by dilution with 710 µL of MilliQ water. Citrate standards with citrate concentrations of 0.01-0.50 mM required for quantification were prepared by dissolving sodium citrate tribasic dihydrate in MilliQ water, followed by a dilution step and addition of the same amount of concentrated HCl as used for NM dissolution. The calibration curves were always measured on a daily basis prior to sample measurements.

For the PyGC-MS measurements, a gas chromatograph (GC, 7890B, Agilent Technologies) with a mass selective detector (MSD, 5977B, Agilent Technology) was equipped with a micro-furnace doubleshot pyrolyzer (PY3030iD, Frontier Laboratories). The pyrolyzer was connected to the GC via a split/splitless interface. The interface was kept at 300 °C and operated in split mode with a ratio of 1:30. An Ultra Alloy 5 capillary column (l = 30 m, iD = 0.25 mm, film thickness = 0.25 µm) was used in the GC with a helium flow of 1 mL/min. The temperature program consisted of 120 s at 40 °C, followed by a rise at 10 °C/min to 300 °C, followed by a 10 min hold at 300 °C. The MSD’s electron impact (EI) ionization energy was 70 eV and the scan range 15–550 amu. Mass spectra assignment was done by comparison with the NIST14 mass spectrometry (MS) library. Solid/powder samples were weighed into 80 µL crucibles using a microbalance (Sartorius CP2P). Sodium citrate tribasic dihydrate was ground with an agate mortar and pestle to reduce particle size. Only small particles allow for the weighing of very small masses. Sodium citrate tribasic dihydrate has a calculated citrate content of 64.3%. The expected citrate content for the NM was below 15%. Sample mass was chosen with respect to the expected citrate content and varied between 10 and 1300 µg. Crucibles were dropped in the pyrolyzer and pyrolyzed in a helium atmosphere at a temperature of 600 °C. Quantification of citrate in the previously dried NM samples by PyGC-MS was carried out based on acetone release, which is one of citrate’s pyrolysis products, using sodium citrate tribasic dihydrate as a calibration standard. Acetone was quantified via the peak area in the measured selective ion current (SIC) for m/z = 58 (molecule ion of acetone) at 1.57 min.

All calculations were done using a molar mass of citrate of 189 g/mol. Unless otherwise stated, the reported uncertainties are standard deviations (SDs) derived from replicate measurements (n = 2 or 3).

## Results and discussion

Ligand exchange is a common strategy to modify the surface of NMs and to tune their colloidal stability and dispersibility in different media. However, it is challenging to achieve and confirm the quantitative removal of the initial ligand(s) present from NM synthesis and ligand replacement with a new one. In many cases, the ligand exchange process is expected to be not quantitative, leaving small amounts of the original ligand or ligand mixtures, or intermediate species, adsorbed onto the NM surface, such as NOBF_4_ utilized for ligand removal for UCNPs, or solvent molecules, such as DMF. Depending on the analytical method used for surface analysis and its chemo-selectivity, such residual species can interfere with the quantification of newly introduced ligands, for example, by contributing to the overall organic content as measured by TGA. This renders the comparison of results obtained with different analytical methods, which differently respond to such possible sources of uncertainty, crucial for accurate NM surface analysis and reliable ligand quantification.

### Quantification of citrate surface ligands on UCNPs using RP-HPLC and TGA

#### Characterization of UCNPs

**Fig. 2:**
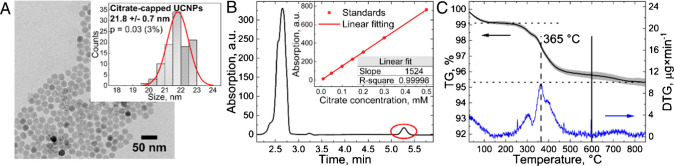
**A** TEM image and size distribution (inset) of the citrate-capped UCNPs; **B** RP-HPLC chromatogram of the citrate-capped UCNPs dissolved in HCl; the inset shows the calibration curve employed for quantifying the citrate amount by photometric citrate detection at 210 nm; **C** TG (black, left axis) and DTG (blue, right axis) curves obtained for the citrate-capped UCNPs

To verify the versatility of our RP-HPLC method with photometric detection for quantifying coordinatively bound citrate surface ligands that has been recently demonstrated for IONPs [41], in this study, we chose another class of NMs, increasingly applied in the material and life sciences, luminescent UCNPs prepared as described in the experimental section. The citrate-capped UCNPs obtained by ligand exchange are characterized by a narrow size distribution (polydispersity < 5%) with an average size of 21.8 nm as derived from TEM (Fig.  2A). DLS measurements confirm the absence of NP aggregation (SI, Fig. S1). The negative zeta potential (−24 mV) supports successful ligand exchange.

#### Citrate quantification on UCNPs

A representative RP-HPLC chromatogram of citrate-capped UCNPs dissolved in HCl (Fig. 2B), obtained with our previously established RP-HPLC method [41], shows a well-defined peak at a retention time of about 5.3 min corresponding to citrate [51]. This peak does not overlap with other peaks originating from unknown impurities and species, e.g., from UCNP synthesis. Also, it is well-separated from the peak at about 2.5 min, which corresponds to ionic species such as chloride ions and/or iron species released in solution after NP dissolution, that pass through the RP-HPLC column without retention. The calibration curve (Fig. 2B, inset), measured with seven standards containing known concentrations of sodium citrate tribasic dihydrate (SI, Fig. S2) in the concentration range of 0.01 mM to 0.50 mM, can be fitted with a linear regression. According to RP-HPLC analysis, the citrate concentration in the dissolved UCNP sample equals 0.102 mM corresponding to 1.134 mM in the stock UCNP dispersion. This yields 4.25 ± 0.09 wt% considering the mass concentration of the stock dispersion (5.04 ± 0.10 mg/mL), which was gravimetrically determined.

To validate the obtained result, we performed a TGA analysis of the dried citrate-capped UCNPs. TGA non-selectively provides the total amount of organic substances present in the sample released at defined temperatures from a previously weighted sample, which does not decompose at the chosen temperatures. The TG curve obtained for the dried citrate-capped UCNPs (Fig.  2C) exhibits an overall mass loss of about 4.7 wt%, including 0.9 wt% attributed to adsorbed water. Analysis of the TGA data yields a citrate-associated mass loss of 3.8 ± 0.4 wt% at elevated temperatures of 150-850 °C, with the main decomposition step observed at 365 °C according to the DTG curve. This value closely matches the result obtained by RP-HPLC, considering the typical measurement uncertainty of this method of about 10%. This suggests that the total organic content of the UCNP sample mostly originates from citrate and confirms a complete exchange of oleate ligands for citrate.

### PyGC-MS measurements of citrate on UCNPs

**Fig. 3:**
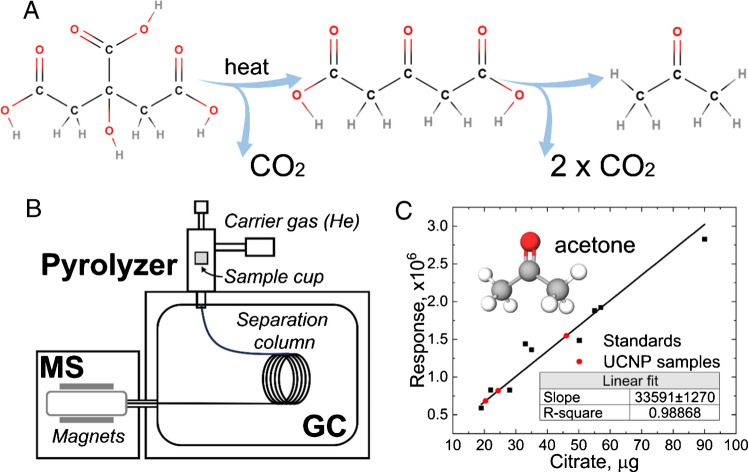
**A** Scheme of the thermal decomposition of citric acid; **B** Scheme of the PyGC-MS setup used; **C** Calibration curve used for quantifying the amount of UCNP citrate surface ligands by PyGC-MS from the amount of released acetone

In a PyGC-MS measurement, a known amount of an otherwise non-volatile sample is thermally cracked in a pyrolysis oven under inert gas to produce volatile products. The oven is directly connected to a gas chromatography setup, where the volatile species are separated upon interaction with the chosen column. The eluates are continuously fed in a gas stream into a mass spectrometer for species analysis and identification. Thereby, PyGC-MS exploits the resolving power and identification capabilities of a GC-MS instrument for originally non-GC accessible samples. Citric acid thermally decomposes to acetone dicarboxylic acid (ADA), releasing carbon dioxide (CO_2_). Decarboxylation of ADA then results in the formation of acetone and the release of two CO_2_ molecules (Fig.  3A) [52]. Subsequently, the volatile decomposition products such as acetone and CO_2_ are effectively detected and quantified by GC-MS. For citrate quantification, the PyGC-MS setup shown in Fig. 3B was employed using a pyrolysis temperature of 600 °C, which corresponds to the plateau region observed in the TG profile of the citrate-capped UCNPs and the minimum of the DTG curve (solid line in Fig.  2C). The PyGC-MS chromatogram of sodium citrate tribasic dihydrate used as a calibration standard (SI, Fig. S3) reveals two strong bands at about 1.34 min and 1.57 min, which correspond to CO_2_ and acetone, respectively. While the CO_2_ signal showed a linear response to citrate concentration only in a limited range of citrate masses up to about 30 µg, we observed a linear trend for the acetone signal over the entire range of the citrate masses tested (SI, Fig. S4A). Therefore, acetone was chosen as the target analyte for the quantitative analysis of the amount of citrate ligands bound to the UCNPs. The method’s robustness was determined by the comparison of calibration curves measured at different times (1 year apart) with the PyGC-MS setup, varying by approximately 8% (SI, Fig. S4B). For both calibrations, the linearity was confirmed for the citrate mass range from 20 µg to 100 µg with R^2^ > 0.98.

According to the PyGC-MS measurements of the citrate-capped UCNPs (SI, Fig. S5), the citrate amount was determined to be 3.3 ± 0.3 wt% from triplicate measurements. This gives a standard deviation of the UCNPs measurements of about 9%. Main factors contributing to the calibration uncertainty are associated with the challenging weighing and handling of very small sample masses in the range of ten µg and the intrinsic variability of the PyGC-MS instrument, i.e., the repeatability of the PyGC-MS signals. To estimate the uncertainty of the calibration (Fig.  3C), the response per µg of citrate was calculated for each calibration measurement. These response factors contain uncertainties from both the determination of the sample mass and the PyGC-MS instrument itself. The standard deviation of these response factors within the calibration range is 13%, which is considered a good estimation of the calibration uncertainty. By combining the uncertainty from the UCNPs measurements of 9% with the uncertainty of the calibration of 13%, using the square root of the sum of the squares of the respective uncertainties, we estimated the overall uncertainty of citrate quantification by PyGC-MS to about 16% (SI, Eq. 1). This value is consistent with literature reports, which reveal uncertainties ranging from 5 to 20% for analyte quantification with PyGC-MS [45]. Considering the overall uncertainty, the citrate concentration in the citrate-capped UCNP sample obtained by PyGC-MS closely matches the values determined by RP-HPLC and TGA.

### Influence of NM type on citrate quantification by PyGC-MS

#### Characterization of IONPs

To assess the applicability of the PyGC-MS method for quantifying citrate surface ligands coordinated to other types of NMs, also citrate-capped IONPs were investigated. Therefore, the citrate-capped IONPs were prepared using the same two-step ligand exchange procedure as employed before for the UCNPs. TEM revealed an average particle size of 9.5 nm with a narrow size distribution, and DLS measurements confirmed the absence of particle aggregation in the aqueous dispersion (SI, Fig. S6). The negative zeta potential (−68 mV) supported the successful surface functionalization with citrate.

#### Citrate quantification on IONPs

**Fig. 4:**
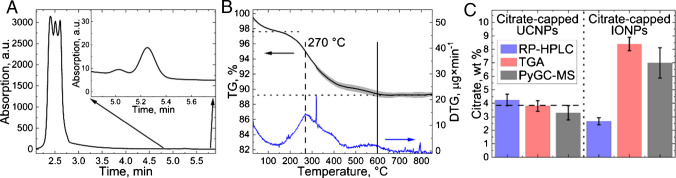
**A** RP-HPLC chromatogram of the citrate-capped IONPs dissolved in HCl, the inset shows the peak corresponding to citrate detected at 210 nm; **B** TG (black, left axis) and DTG (blue, right axis) curves of the citrate-capped IONPs; **C** Bar diagram displaying the citrate concentration obtained in the citrate-capped UCNPs and IONPs samples using RP-HPLC, TGA, and PyGC-MS

RP-HPLC measurements of the citrate-capped IONPs dissolved in HCl (Fig.  4A) yielded a citrate amount of 2.67 ± 0.03 wt%, calculated from the measured citrate concentration of 0.063 mM and the gravimetrically determined mass concentration of 4.95 ± 0.04 mg/mL of the stock dispersion. TGA measurements revealed a total mass loss of about 10.8 wt% (Fig. 4B) with a contribution of adsorbed water by about 2.4 wt%. The remaining mass loss of approximately 8.4 ± 0.5 wt% is attributed to the decomposition of the organic content of the IONP sample, including citrate, with the main degradation step occurring at 270 °C. Astonishingly, this degradation temperature is considerably lower than the degradation temperature of 365 °C observed for the citrate-capped UCNP sample. We tentatively attribute the observed difference in the main decomposition temperatures to the catalytic activity of the IONPs [53, 54], which can promote the degradation of citrate. The higher organic content derived from the TGA measurements compared to the more chemo-selective RP-HPLC analysis suggests the presence of additional organic residues such as oleate or DMF, remaining after the ligand exchange process on the surface of IONPs. These results agree well with the previously reported analysis of citrate-capped IONPs prepared using a different ligand exchange route [41].

PyGC-MS measurements of the citrate-capped IONPs (SI, Fig. S7), performed under the same conditions as used before for the citrate-capped UCNPs, revealed a citrate concentration of 7.0 ± 0.6 wt%. This value considerably exceeds the amount of citrate measured by RP-HPLC, as is highlighted in Fig. 4C. This figure summarizes the results of citrate quantification for the UCNP and IONP samples as determined by RP-HPLC, TGA, and PyGC-MS, considering the respective method-specific uncertainties of 10% and 16% for RP-HPLC and PyGC-MS, respectively. For the citrate-capped UCNPs, all three analytical techniques give closely matching results, indicating nearly complete ligand exchange and confirming the validity of the different quantification approaches. For the citrate-capped IONPs, however, the PyGC-MS method overestimates the citrate amount compared to the results of the chemo-selective RP-HPLC method. This discrepancy most likely originates from our assumption that the decomposition of citrate coordinated to NPs occurs following the same reaction pathway as the sodium citrate tribasic dihydrate used as a standard for citrate quantification. However, IONPs, which are known to be catalytically active, could encourage citrate decomposition [53, 54], leading to an increased formation of acetone at 600 °C. This hypothesis is supported by the lower decomposition temperature observed for the citrate-capped IONP sample in the DTG curve, which indicates a catalytically enhanced citrate degradation on the surface of the IONPs.

### Assessing a possible influence of the ligand exchange procedure on citrate quantification

#### Characterization of IONPs-2

**Fig. 5:**
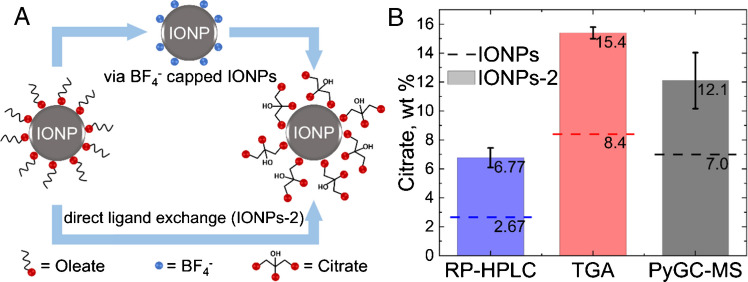
**A** Scheme of the two ligand exchange procedures used for IONPs; **B** Bar diagram displaying the citrate concentration obtained in the citrate-capped IONPs samples prepared by two-step (IONPs, dashed lines) and direct (IONPs-2, columns) ligand exchange procedures using RP-HPLC, TGA, and PyGC-MS

Next, we explored a possible influence of the ligand exchange procedure on our PyGC-MS method for IONPs. Therefore, we used the same ligand exchange protocol for IONPs as applied for the citrate-capped IONPs prepared for the development of the RP-HPLC method [41]. This workflow allows for direct ligand exchange from oleate to citrate at an elevated temperature of 100 °C, in comparison to the two-step NOBF_4_ method utilized before, which was adopted from a typical UCNP phase transfer procedure employing citrate (Fig. 5A). The ligand exchange was monitored by TEM, DLS, and zeta potential measurements (SI, Fig. S8A-B). The hydrodynamic size of the citrate-capped IONPs-2 of 9 nm and the zeta potential of −44 mV confirmed the absence of agglomerated IONPs in the aqueous dispersion and successful ligand exchange.

#### Citrate quantification on IONPs-2

Figure 5B summarizes the results of citrate quantification from RP-HPLC, TGA, and PyGC-MS measurements (SI, Fig. S8C-D and Fig. S9) for citrate-capped IONPs prepared by two-step (IONPs, dashed lines) and direct (IONPs-2, columns) ligand exchange procedures. The results of RP-HPLC and TGA measurements obtained for the citrate-capped IONPs-2 were consistent with previously reported data, confirming the reproducibility of the direct ligand exchange approach [41]. RP-HPLC, as a more chemo-selective method, revealed a higher citrate content for this sample compared to the citrate-capped IONPs obtained by the two-step ligand exchange procedure. This suggests that the direct ligand exchange procedure should be more efficient for the surface functionalization of IONPs with citrate, leading to higher citrate coverage than the two-step process. The PyGC-MS results for the citrate-capped IONPs-2 sample exhibited the same trend as observed before for IONPs, namely an apparent increase in citrate content compared to the values determined by RP-HPLC. It further supports our hypothesis that the overestimation of citrate content in PyGC-MS measurements originates from the catalytic activity of iron oxide during the pyrolysis process. Since the citrate-capped IONPs-2 sample contains a higher citrate amount, the iron oxide-to-citrate ratio in this sample is lower compared to two-step citrate-capped IONPs. The lower relative amount of catalytically active iron oxide could explain the reduced difference between the PyGC-MS and RP-HPLC. This assumption is also supported by the absence of the last mass loss step at about 800 °C in the TGA data obtained for two-step citrate-capped IONPs, which indicates a more pronounced catalytic enhancement of the citrate decomposition for the IONPs sample in contrast to the IONPs-2 sample.

An overview of the analytical information and results of the citrate quantification obtained by the methods utilized in this study is summarized in Table 1 and in the SI in Table S1. This comparison demonstrates that the previously developed RP-HPLC approach provides accurate and citrate-selective measurements when applied to UCNPs, consistent with its performance reported for IONPs in previous work. In addition, the newly assessed PyGC-MS method, while exhibiting some NM-specific limitations, emerges as a promising tool for surface ligand determination due to the simpler sample preparation workflow compared to RP-HPLC and the substantially reduced sample amount required compared to TGA. However, the applicability of this method, explored in this proof-of-concept study for the first time for surface modified NPs, must be further explored with different types of nanomaterials for method optimization and to determine material-specific limitations.

**Table 1:** Overview of the employed methods for citrate quantification

Parameter	RP-HPLC	PyGC-MS	TGA
Measurand	Citrate absorption	Acetone release	Mass loss
Selectivity for citrate	Selective	Semi-selective	Non-selective
Sample preparation	Dissolution of NPs	Drying	Drying
Calibration standards	Solutions with different citrate concentration	Solids with different citrate mass	-
Tested linear range	0.01-0.50 mM	20-100 µg	-
Limit of citrate quantification	3 µg/mL [51]	~ 20 µg*	-
Average used NPs sample amount per measurement, mg	0.5	0.8	3

*The lowest tested citrate amount, limit of citrate quantification was not determined

## Conclusion and outlook

In summary, we could demonstrate the versatility of our reversed-phase high-performance liquid chromatography (RP-HPLC) method with photometric detection, developed for quantifying citrate ligands on iron oxide nanoparticles (IONPs), for the determination of citrate on different types of nanomaterials (NMs) as demonstrated in this study for upconversion nanoparticles (UCNPs). This chemo-selective method provides reproducible and accurate results for samples of dissolved NMs, quantitatively releasing surface-coordinated citrate ligands into solution. Pyrolysis-gas chromatography-mass spectrometry (PyGC-MS) enables the direct analysis of dried NM samples without the need for NM dissolution or other sample preparation steps, and for a considerably reduced sample amount compared to thermogravimetric analysis (TGA), but its measurement uncertainty of 16% exceeds the typical uncertainties of the RP-HPLC method and TGA of about 10%. Although PyGC-MS is chemo-selective, the reliability of the indirect quantification of the surface ligand by measuring a decomposition product relies on the assumption of an identical decomposition behaviour of the ligands on NM surface and the calibration standard used for quantification. This was demonstrated in our multimethod study of citrate-capped UCNPs and IONPs by cross-comparison of RP-HPLC, TGA, and PyGC-MS results. The good match obtained for citrate-capped UCNPs and the citrate overestimation observed for citrate-capped IONPs underline the principal applicability of PyGC-MS for NM surface analysis together with the need to carefully consider the chemical nature of the NM sample, particularly a possible catalytic activity. The latter can affect the decomposition behaviour of NM surface ligands and result in an increase in the amount of decomposition products formed and measured at certain temperatures, thereby favouring an overestimation of the ligand amount.

Our findings highlight the importance of characterizing the surface chemistry of NMs with different analytical methods, relying on different signal generation principles and sample preparation workflows and addressing different, yet correlated measurands. This simplifies method validation and the identification of method-inherent limitations and method-specific effects, as prerequisites for a reliable and accurate surface analysis. Thereby, additional information on the application-relevant physicochemical properties of NMs can also be gained, as recently demonstrated by us in a multimethod study of a large series of aminated silica nanoparticles using quantitative nuclear magnetic resonance technique, optical assays, X-ray photoelectron spectroscopy, and a potentiometric titration method [17]. Despite some limitations identified in this study, PyGC-MS presents an attractive chemo-selective tool for determining NM surface ligands, although future studies of application-relevant NMs with varying catalytic activity with RP-HPLC, PyGC-MS, and TGA are needed to provide more in-depth insights into material-specific limitations of PyGC-MS and to optimize PyGC-MS workflows. Thereby, also criteria for the fast identification of catalytic effects affecting ligand quantification can be identified, such as a reduced degradation temperature obtained by TGA. In addition, the suitability of PyGC-MS for NM aging and screening studies will be assessed.

## Supplementary Information

Below is the link to the electronic supplementary material.

Supplementary file1 (DOCX 2.16 MB)

## Data Availability

Data are available upon request to the corresponding author.
