# Supplementary material for: Cross-validation of analytical methods for citrate ligand quantification on upconversion and iron oxide nanoparticles
Source: Anal Bioanal Chem. 2026 Mar 4;418(16):5181–91. doi: 10.1007/s00216-026-06402-8 (PMC13424209; doi:10.1007/s00216-026-06402-8)
Supplement: Supplementary file 1 — Supplementary file1 (DOCX 2.16 MB) [file 216_2026_6402_MOESM1_ESM.docx]

Supporting Information (SI)

Cross-Validation of Analytical Methods for Citrate Ligand Quantification on Upconversion and Iron Oxide Nanoparticles

Anna Matiushkina^1,2^, Sven Brehme^3^, Isabella Tavernaro^1^, Elina Andresen^1^, Sarah-Luise Abram^1^, Matthias Koch^4^, and Ute Resch-Genger^1,*^

^1^Division *Biophotonics*, Bundesanstalt für Materialforschung und -prüfung (BAM), Richard-Willstaetter-Straße 11, 12489 Berlin, Germany;

^2^Department of Biology, Chemistry, and Pharmacy, Free University Berlin, Arnimallee 22, 14195 Berlin, Germany

^3^Division *Technical Properties of Polymeric Materials*, Bundesanstalt für Materialforschung und -prüfung (BAM), Unter den Eichen 87, 12205 Berlin, Germany

^4^Division *Organic Trace and Food Analysis*, Bundesanstalt für Materialforschung und -prüfung (BAM), Richard-Willstaetter-Straße 11, 12489 Berlin, Germany

*corresponding author: Ute Resch-Genger; email: [ute.resch@bam.de](mailto:ute.%20resch@bam.de)

Contents

[1. Experimental Information 2](#_Toc211861769)

[2. Nanoparticle characterization and citrate quantification 3](#_Toc211861770)

[3. References 6](#_Toc211861771)

1. Experimental Information

*Synthesis of UCNPs & ligand exchange.* Co-doped oleate-capped upconversion nanoparticles NaYF_4_:Yb^3+^,Er^3+^ (UCNPs) were synthesized following a procedure from Wilhelm et al. for the large-scale synthesis of hexagonal-phase UCNPs [1]. The resulting UCNPs were dispersed in cyclohexane and stored at 4 °C. For ligand exchange, at the first step the oleate-capped UCNPs were dispersed in cyclohexane at a concentration of approximately 10 mg/mL. An equal volume of dimethylformamide (DMF) was added to the dispersion of UCNPs in cyclohexane to create a biphasic system. NOBF₄ (2 mass-eq. of used UCNPs) was then introduced into the mixture. The reaction mixture was stirred for around 60 min at room temperature (rt). As a result, UCNPs transferred from the nonpolar cyclohexane phase into the polar DMF phase, were collected by centrifugation from the DMF phase and washed twice with DMF. At the second step, an aqueous solution of trisodium citrate dihydrate (2 mass-eq. of used UCNPs) was added to UCNPs redispersed in DMF. The mixture was stirred for 1 hour at rt, allowing the citrate molecules to adsorb onto the nanoparticle surface via their carboxylate groups. The citrate-capped UCNPs were collected by centrifugation, washed twice with water, finally dispersed in MilliQ water, and stored at 4 °C with concentration of 5.04 ± 0.10 mg/mL.

*Synthesis of IONPs & ligand exchange.* The iron oxide nanoparticles (IONPs) were synthesized following the procedure from Park et al. [2], using a thermal decomposition of iron oleate in 1-octadecene with oleic acid at 320 °C under an argon flow. To exchange oleate for citrate ligands, the procedure similar to UCNPs ligand exchange was used with minor changes. The resulting oleate-capped IONPs were dispersed in cyclohexane at a concentration of approximately 10 mg/mL. An equal volume of DMF containing NOBF₄ (1 mass-eq. of used IONPs) was added to the dispersion of IONPs, and the mixture was stirred for around 90 min at rt. Then, chloroform was added to IONPs in DMF phase, and IONPs were collected by centrifugation and redispersed in DMF. Later, obtained IONPs in DMF were mixed with an aqueous solution of sodium citrate tribasic dihydrate (1.3 mass-eq. of used IONPs) and stirred at rt overnight. The citrate-capped IONPs were collected by centrifugation, washed twice with water, finally dispersed in MilliQ water, followed by filtering with a 0.2 μm syringe filter (CHROMAFIL Xtra H-PTFE, Macherey-Nagel), and stored at rt with concentration of 4.95 ± 0.04 mg/mL.

For direct ligand exchange procedure [3], the oleate-capped IONPs were dispersed in 1,2 dichlorobenzene at a concentration of approximately 5 mg/mL. An equal volume of DMF containing citric acid (0.9 mass-eq. of used IONPs) was added to the dispersion of IONPs, and the mixture was stirred at 100 °C for 24 h. Then, IONPs were collected by centrifugation and washed using diethyl ether, acetone, and water. The resulting citrate-capped IONPs (IONPs-2) were dispersed in MilliQ water with addition of NaOH solution and stored at a pH of 7.9 at rt with concentration of 5.08 ± 0.07 mg/mL.

2. Nanoparticle characterization and citrate quantification

| 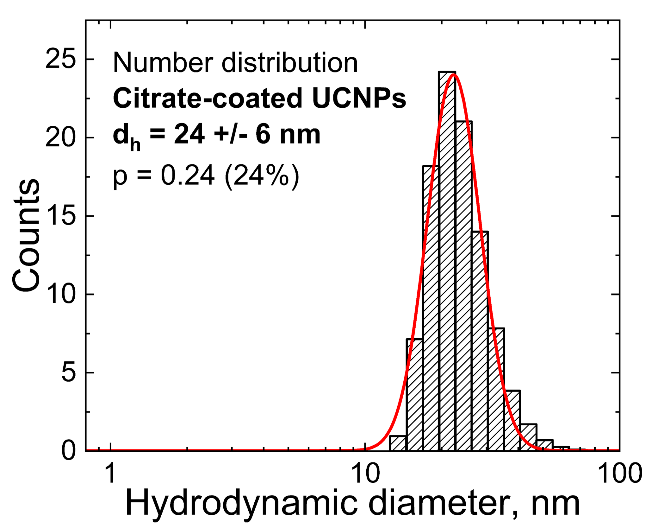 |
| --- |
| Figure S1. Number-based size distribution of the citrate-capped UCNPs as obtained by DLS measurements |

| 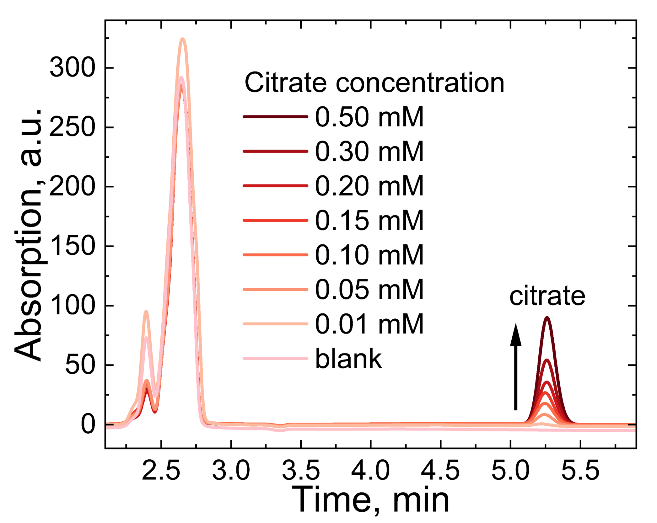 |
| --- |
| Figure S2. RP-HPLC chromatograms of the standard solutions containing different citrate concentrations |

| 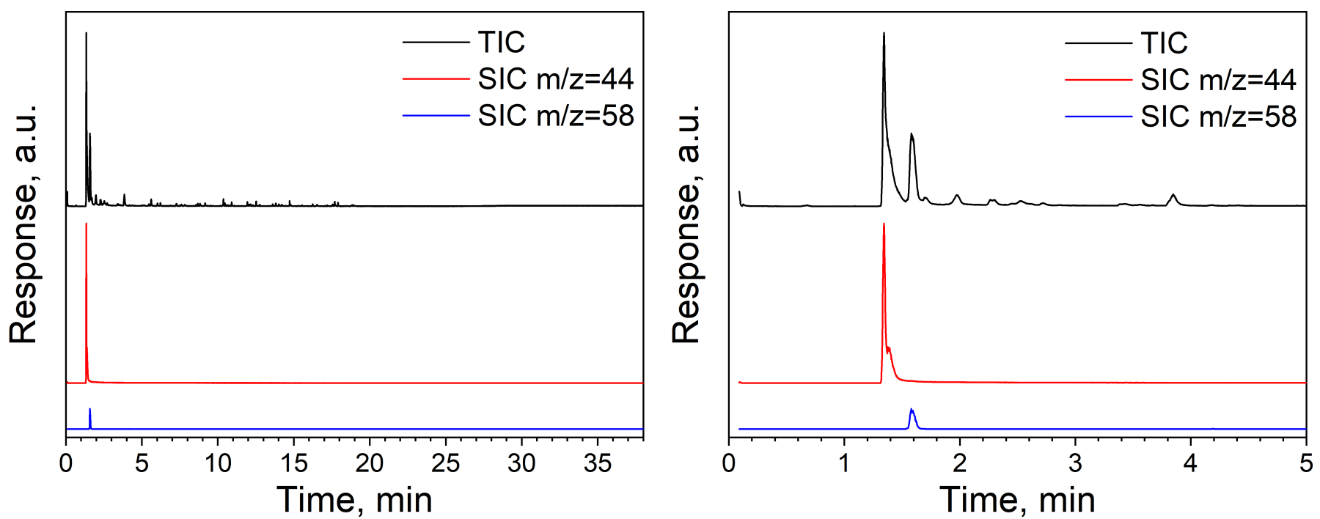 |
| --- |
| Figure S3. PyGC-MS chromatogram of sodium citrate tribasic dihydrate, full (left) and first 5 min (right), total ion current (TIC) and selective ion current (SIC) for m/z=44 (CO_2_) and m/z=58 (acetone) |

| 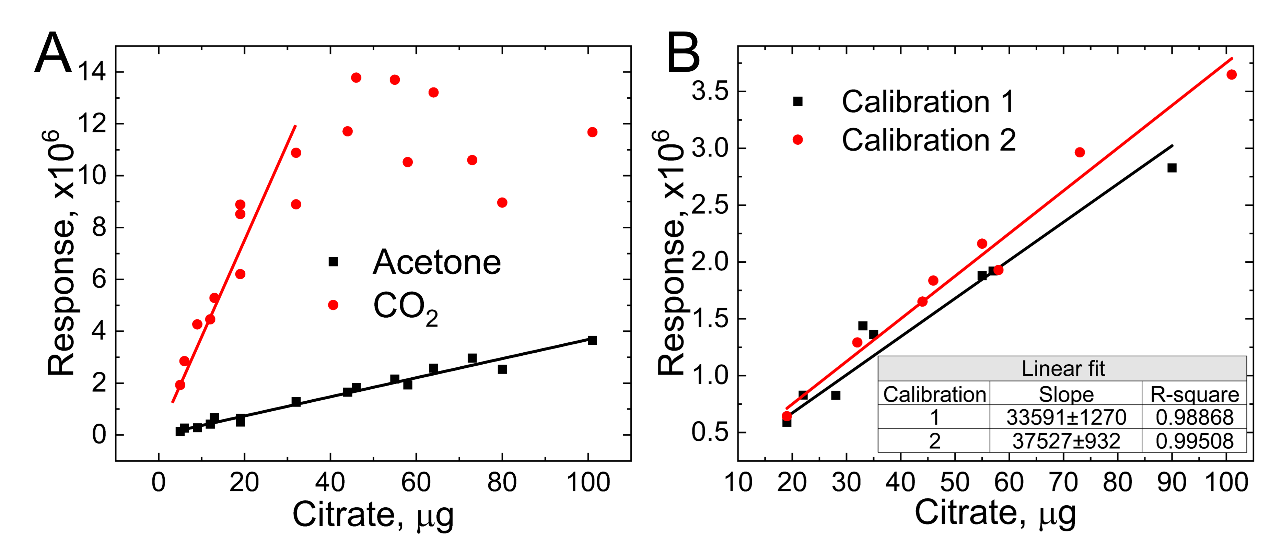 |
| --- |
| Figure S4. (A) Dependences of the acetone and CO_2_ signals from the PyGC-MS data on the citrate mass; (B) Calibration curves based on acetone release measured by PyGC-MS method on different time frames |

| 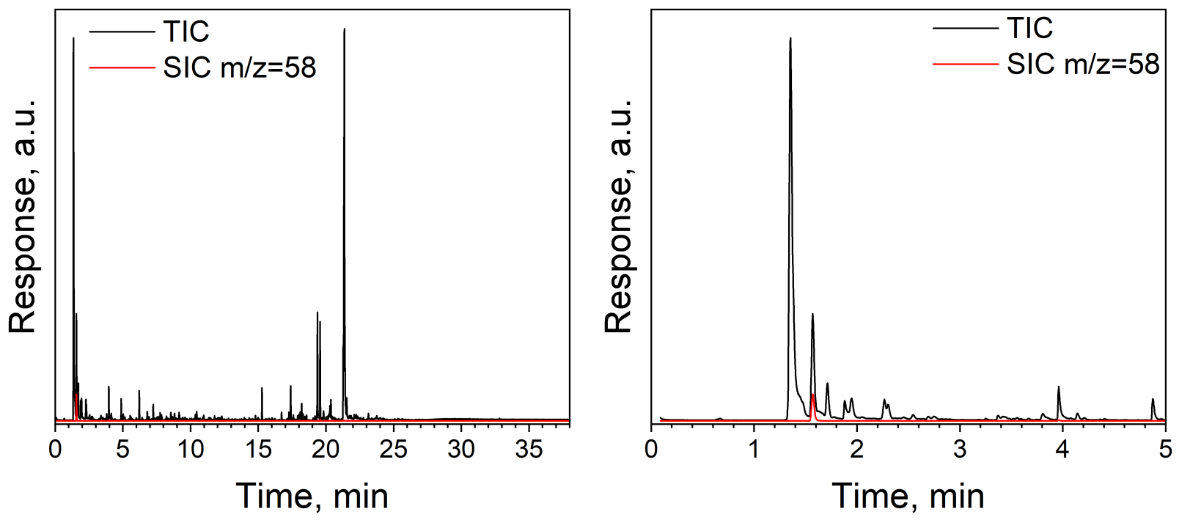 |
| --- |
| Figure S5. PyGC-MS chromatogram of citrate-capped UCNPs, full (left) and first 5 min (right), total ion current (TIC) and selective ion current (SIC) for m/z=58 (acetone) |

The overall uncertainty of citrate quantification by PyGC-MS:

$u_{PyGC-MS}=\sqrt[2]{u_{UCNPs}^{2}+u_{cal}^{2}}= \sqrt[2]{9^{2}+{13}^{2}}=16 \%$ (1)

where $u_{UCNPs}$ is the uncertainty of the UCNP measurements and $u_{cal}$ the uncertainty of calibration.

| 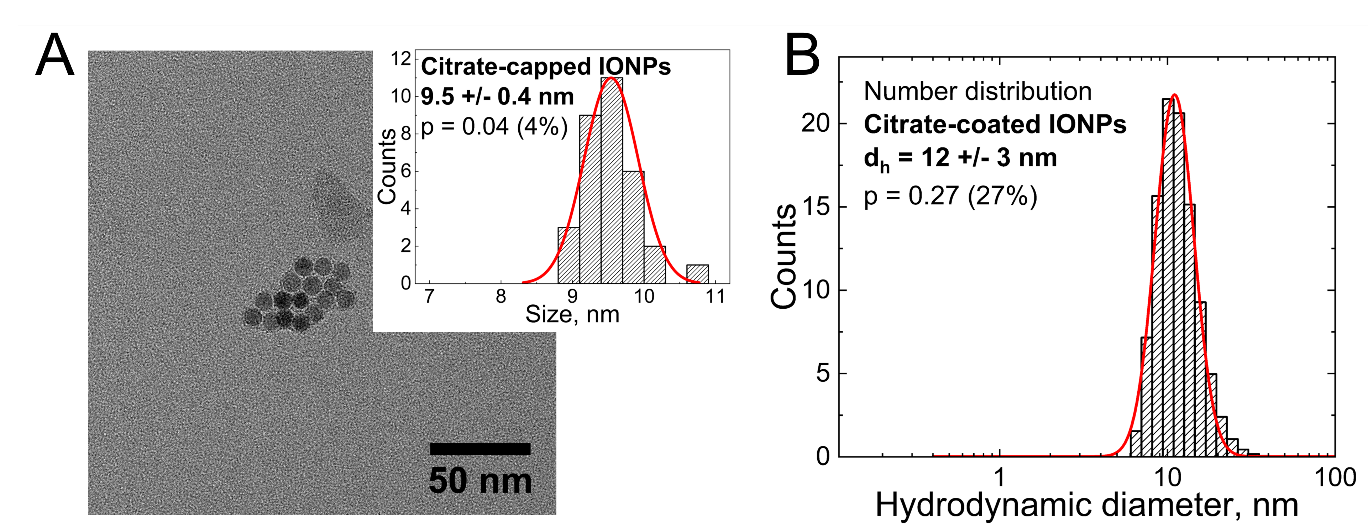 |
| --- |
| Figure S6. (A) TEM image and size distribution (inset) of the citrate-capped IONPs; (B) Number-based size distribution of the citrate-capped IONPs as obtained by DLS measurements |

| 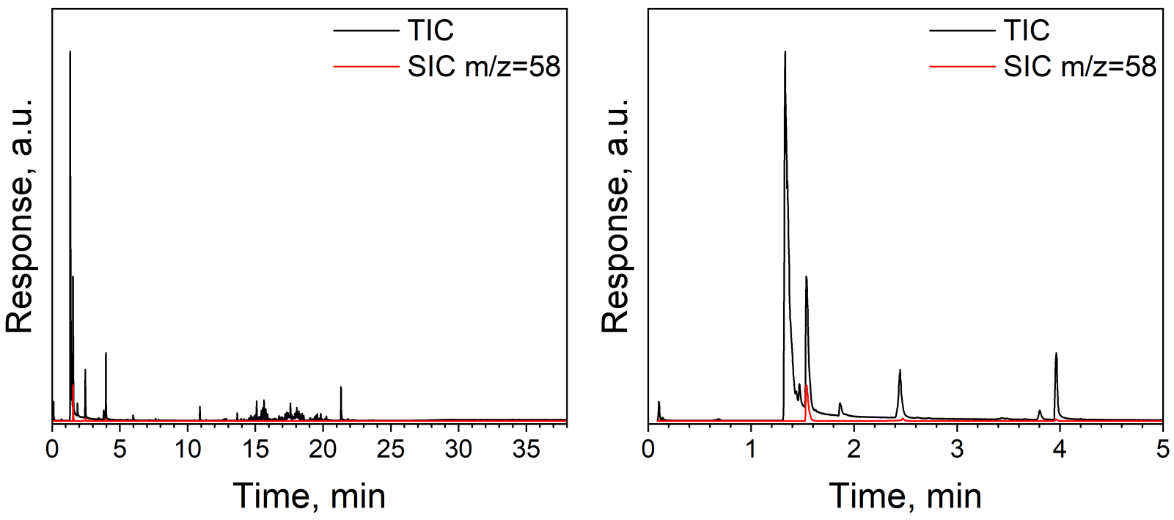 |
| --- |
| Figure S7. PyGC-MS chromatogram of citrate-capped IONPs, full (left) and first 5 min (right), total ion current (TIC) and selective ion current (SIC) for m/z=58 (acetone) |

| 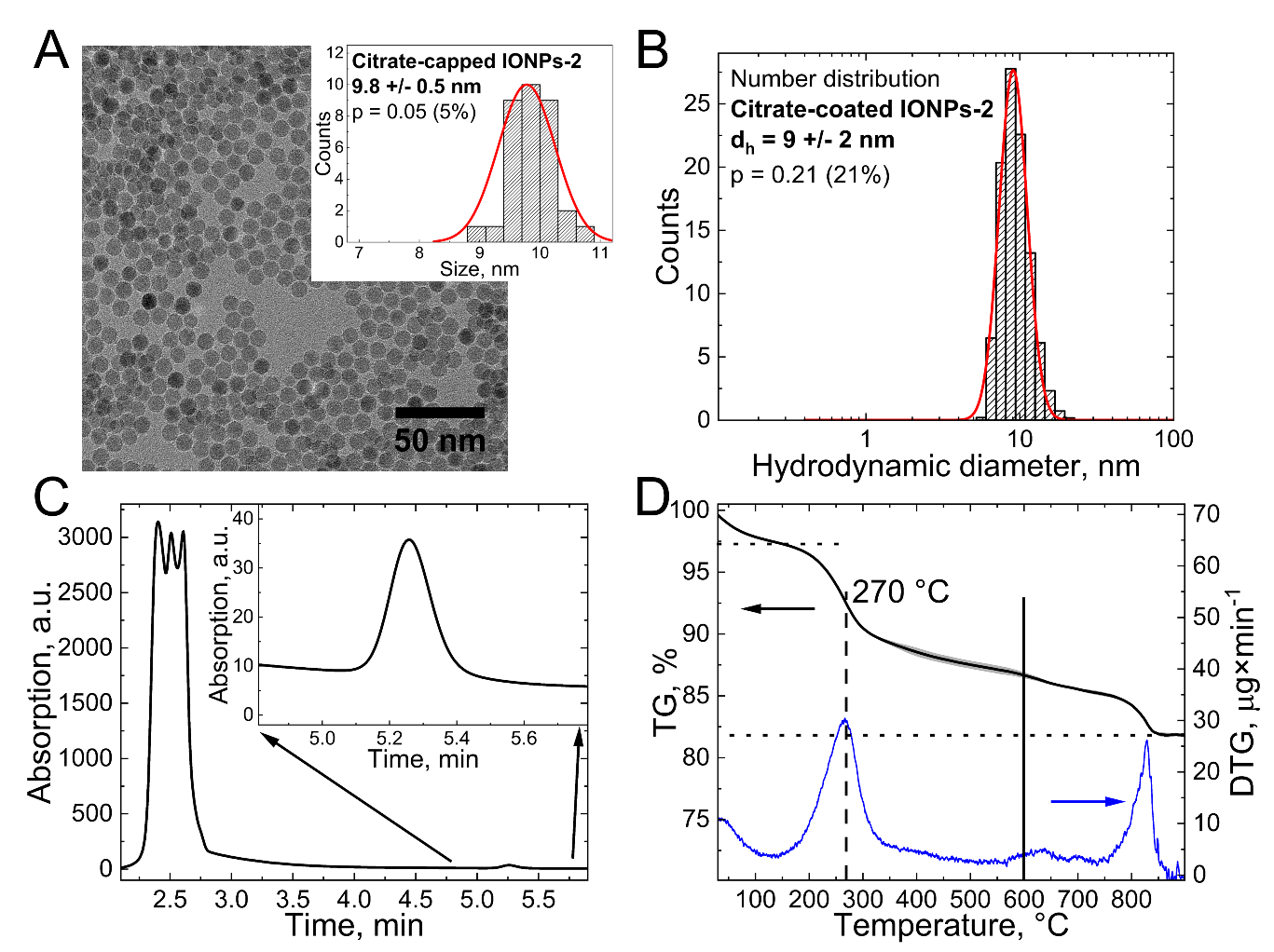 |
| --- |
| Figure S8. (A) TEM image and size distribution (inset) of the citrate-capped IONPs-2; (B) Number-based size distribution of the citrate-capped IONPs-2 as obtained by DLS measurements; (C) RP-HPLC chromatogram of the citrate-capped IONPs-2 dissolved in HCl; the inset shows the peak corresponding to citrate detected at 210 nm; (D) TG (black, left axis) and DTG (blue, right axis) curves of the citrate-capped IONPs-2 |

| 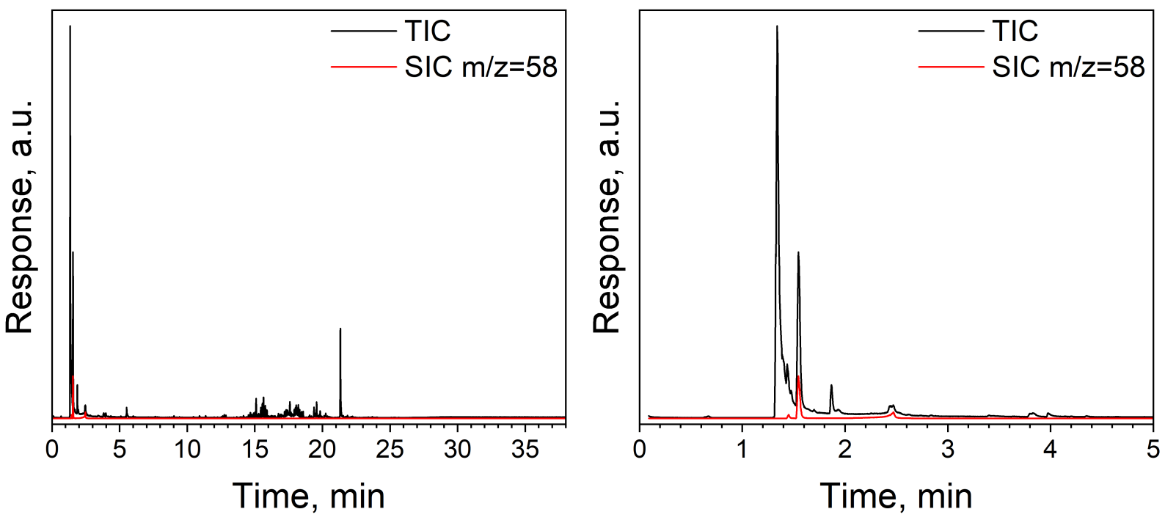 |
| --- |
| Figure S9. PyGC-MS chromatogram of citrate-capped IONPs-2, full (left) and first 5 min (right), total ion current (TIC) and selective ion current (SIC) for m/z=58 (acetone) |

**Table S1.** Results of citrate quantification obtained by the different analytical methods

| **Citrate content** | **RP-HPLC** | **PyGC-MS** | **TGA*** |
| --- | --- | --- | --- |
| UCNPs sample [wt%] | 4.25 ± 0.09 | 3.3 ± 0.3 | 3.8 ± 0.4 |
| IONPs sample [wt%] | 2.67 ± 0.03 | 7.0 ± 0.6 | 8.4 ± 0.5 |
| IONPs-2 sample [wt%] | 6.77 ± 0.11 | 12.1 ± 1.4 | 15.4 ± 0.3 |

* Total organic content of the sample

# 3. References

1. Wilhelm S, Kaiser M, Würth C, Heiland J, Carrillo-Carrion C, Muhr V, et al. Water dispersible upconverting nanoparticles: effects of surface modification on their luminescence and colloidal stability. Nanoscale. 2015;7(4):1403–10.

2. Park J, An KJ, Hwang YS, Park JG, Noh HJ, Kim JY, et al. Ultra-large-scale syntheses of monodisperse nanocrystals. Nat Mater. 2004;3(12):891–5.

3. Monnier CA, Crippa F, Geers C, Knapp E, Rothen-Rutishauser B, Bonmarin M, et al. Lock-In Thermography as an Analytical Tool for Magnetic Nanoparticles: Measuring Heating Power and Magnetic Fields. Journal of Physical Chemistry C. 2017;121(48):27164–75.
